# Whose knowledge counts? Involving communities in intervention and trial design using community conversations

**DOI:** 10.1186/s13063-023-07320-1

**Published:** 2023-06-07

**Authors:** Rochelle A. Burgess, Funmilayo Shittu, Agnese Iuliano, Ibrahim Haruna, Paula Valentine, Ayobami Adebayo Bakare, Tim Colbourn, Hamish R. Graham, Eric D. McCollum, Adegoke G. Falade, Carina King, on behalf of the INSPIRING Project Consortium, Tahlil Ahmed, Samy Ahmar, Christine Cassar, Adamu Isah, Adams Osebi, Abdullahi Magama, Ibrahim Seriki, Temitayo Folorunso Olowookere, Matt McCalla, Obioma Uchendu, Julius Salako, Damola Bakare, Omotayo Olojede

**Affiliations:** 1 Institute for Global Health, University College London, London, UK; 2 Department of Paediatrics, University College Hospital, Ibadan, Nigeria; 3 Department of Global Public Health, Karolinska Institutet, Stockholm, Sweden; 4 Save the Children, Abuja, Nigeria; 5 Save the Children, London, UK; 6 Department of Community Medicine, University of Ibadan, Ibadan, Nigeria; 7 Centre for International Child Health, Murdoch Children’s Research Institute, University of Melbourne, Royal Children’s Hospital, Parkville, Victoria Australia; 8 Department of Pediatrics, School of Medicine, Eudowood Division of Pediatric Respiratory Sciences, Johns Hopkins University, Baltimore, USA; 9 Department of Paediatrics, University of Ibadan, Ibadan, Nigeria

**Keywords:** Community conversations, Trials, Participation, Community engagement, Formative research, Under-5 pneumonia

## Abstract

**Background:**

Current debates in Global Health call for expanding methodologies to allow typically silenced voices to contribute to processes of knowledge production and intervention design. Within trial research, this has typically involved small-scale qualitative work, with limited opportunities for citizens to contribute to the structure and nature of the trial. This paper reports on efforts to move past typical formative trial work, through adaptation of community conversations (CCs) methodology, an action-oriented approach that engages large numbers of community members in dialogue. We applied the CC method to explore community perspectives about pneumonia and managing the health of children under-5 in Northern Nigeria to inform our pragmatic cluster randomised controlled trial evaluating a complex intervention to reduce under-5 mortality in Nigeria.

**Methods:**

We conducted 12 rounds of community conversations with a total of 320 participants, in six administrative wards in Kiyawa Local Government Area, Jigawa state, our intervention site. Participants were male and female caregivers of children under five. Conversations were structured around participatory learning and action activities, using drawings and discussion to reduce barriers to entry. During activities participants were placed in subgroups: younger women (18–30 years of age), older women (31–49 years) and men (18 years above). Discussions were conducted over three 2-h sessions, facilitated by community researchers. Following an initial analysis to extract priority issues and perspectives on intervention structure, smaller focus group discussions were completed with participants in five new sites to ensure all 11 administrative wards in our study site contributed to the design.

**Results:**

We identified enabling and limiting factors which could shape the future trial implementation, including complex power relationships within households and wider communities shaping women’s health decision-making, and the gendered use of space. We also noted the positive engagement of participants during the CC process, with many participants valuing the opportunity to express themselves in ways they have not been able to in the past.

**Conclusions:**

CCs provide a structured approach to deep meaningful engagement of everyday citizens in intervention and trial designs, but require appropriate resources, and commitment to qualitative research in trials.

**Trial registration:**

ISRCTN39213655. Registered on 11 December 2019.

**Supplementary Information:**

The online version contains supplementary material available at 10.1186/s13063-023-07320-1.

## Background

Scholars and activists consistently advocate for the importance of community participation and involvement in health research. For example, Rifkin [1] suggests community contributions to research project design help to ensure that local definitions of problems and contexts are at the heart of interventions. This, alongside community and citizen priority setting, decision-making, planning, problem-solving, implementation, and evaluation are approaches that have been supported by scholars across public health and implementation science domains [2]. The Alma-Ata declaration [3] was the first in a lineage of WHO health frameworks that centred the important role of community engagement for health, with more recent guidelines illuminating the importance of community participation in the achievement of universal health care [4].

Ongoing challenges with the implementation of community health interventions have been linked to research approaches that do not allow for robust or specific definitions of key concepts, such as ‘community’ [5], or ‘participation’ [6]. Furthermore, Global Health spaces have faced criticism for their inability to take community-generated knowledge seriously in research and intervention development [7]. The result of knowledge hierarchies that situate community and local knowledge as inferior to science is the piecemeal adoption of community contribution and reduced opportunities for achieving emancipation and empowerment for improved health [8].

For example, the free maternal and child health programme in Nigeria (FMCHCP) currently being implemented in some states [9] emphasises the role of community-based participatory interventions, designed to support women in taking on and developing skills and knowledge linked to child health [10]. Central to these strategies, is the belief that for community members to take ownership of and improve health outcomes, they must acquire new skills, knowledge and/or motivation rooted in formal health-related knowledge that will enable new forms of action to take place.

Such efforts are examples of the community participation contributes to strengthening health care systems through helping communities with problem-solving skills, encouraging them to take charge of their health and welfare [11], but not on their terms. Evidence suggests that externally imposed knowledge systems can encounter local resistance when pre-existing strengths or cultural processes with the capacity to make positive influences on health, are overlooked or replaced [12]. Research attempts to respond to these tensions through ensuring the meaningful engagement of communities in processes of intervention design, development and evaluation [13]. But how possible is this within the structure of current large-scale evaluation methodologies like trials?

### Whose knowledge counts? Community involvement in gold standard methods for intervention design and evaluation

Trial research remains the gold standard approach for the design and testing of intervention effects. The evolution of trial methods in recent years aligns with attempts to acknowledge the importance of community involvement for health improvement. A recent systematic review of reviews [13] exploring patient involvement in trials identified over 2000 studies where PPI was used in some format within trial research. Within later stages of research pragmatic Randomised Control Trials (RCTs) are increasingly being used to understand the effectiveness of interventions within everyday and routine practice conditions, which has led to novel ways of measuring outcomes that centre community experiences, such as patient-centred outcomes [14]. Adaptive trials, defined by Brown et al. [15] as those open to the modification of trial characteristics based on information from accumulating data, take the real-world focus a step further. However, more open trial methodologies do not automatically create space for meaningful community involvement, despite evidence suggesting equitable involvement of stakeholders in RCT design improves understanding of the cultural contexts of implementation and in securing community ownership after the trial ends [16].

Community-led trials [17] are one approach that more deeply engages with communities across various stages of a trial, including examples of communities being responsible for intervention design and/or delivery. One such example was presented by Lisa Hjelmfors and colleagues [18] who describe the involvement of multiple groups of patients, their relatives and health professionals taking part in co-design of an intervention to improve communication about heart failure trajectory and end-of-life care. Across development and testing phases, “ideas groups” and “prototyping” discussions were facilitated. Ideas groups emphasised understanding of factors that improve communication between patients, relatives and health professionals. Prototyping focused on participant satisfaction with, and reactions to the intervention, and evaluated the intervention potency of being successful among intended users. Findings showed improved knowledge, confidence, and skills to discuss the heart failure trajectory and end-of-life care among patients, relatives, and health professionals. The Camino Verde [19] and Rebuilding from resilience [20] trials are also examples of approaches which centred the involvement of communities around the intervention.

However, not all contexts are suited to community-led trials, as they demand the long-term involvement of participants and a commitment from researchers to invest time and resources to build trust with participants. A more common approach to community involvement in trials is thus the completion of formative research which collects detailed information to aid trial design, including intervention content, delivery mechanisms and implementation, guides the recruitment and retention of study participants, and helps to determine the feasibility and acceptability of an intervention [21, 22]. Recent work by Mannell and colleagues [23] explored the use of qualitative methods within trials and noted the formative phase in RCTs overwhelmingly relies on standard qualitative methods such as focus group discussions, cognitive interviews with community members and establishing and liaising with community advisory groups. However, formative research is often limited by its scale. Small sample sizes common within formative studies mean that our ability to understand the full complexities of contexts and experiences facing potential patients [23]. Advisory groups similarly involve small numbers of participants but are founded in forms of participation that are more consultative than collaborative in scope. In their review of reviews, Price and colleagues [13] noted that participant and patient involvement most often took the shape of gaining feedback on already determined trial design, the moderation of forums or recruiting participants, instead of having an active involvement in research tasks such as study design, analysis or dissemination. This means that communities lack ownership over questions explored, or the future intervention itself. In international development a field closely related to global health, scholars have continually critiqued the dangers of such a form of participation. Susan White [24] identified four forms of participation ranging from nominal (limited power or decision-making) to transformative (with a goal or aim to transfer power to community actors). Modes of participation described by Price and colleagues [13] reflect lower forms of participation where the form and function serves the needs of more powerful external agents, rather than community members.

Is it possible to expand and deepen our engagement with communities at scale during RCT trial design - especially for components such as intervention development? What methods will enable us to normalise more meaningful community engagement at the heart of trial practices? It is at this juncture that we seek to make our contribution. This paper reports on the adaptation of community conversation methodology to involve communities in mapping the contexts that would shape the feasibility and acceptability of participatory interventions to address childhood infectious diseases in Northern Nigeria and planning for trial implementation and intervention delivery.

## Methods: community conversations

Community conversations (CCs) are a powerful tool that encourages critical thinking among participants, stimulates finding tailored solutions to the problem detected, and can lead to lasting community empowerment. Opportunities are created for diverse stakeholders to develop critical consciousness; an awareness of the ways in which relationships between structural contexts and our experiences come about and contribute to the difficulties people face in their everyday lives [25]. Dialogue has been identified as key to processes of social change [26] and the CC method enables community-centred dialogue through a light touch role for the facilitator.

The Nelson Mandela Foundation’s approach to CCs involves five broad stages to generate critical dialogue: Relationship building, Concern Identification, Concern Exploration, Decision making, and Reflection and Review [25]. It was popularised within HIV-related work in Southern Africa and has been used to develop a range of community-led interventions that challenge norms, shift action and provide community-defined solutions to change, including HIV adherence [26] or issues related to gender-based violence [27-29]. However, this approach remains under-utilised.

The CC method is not without its challenges. Given its interest in combining actors across a range of social positions within shared spaces for thinking and action, some evidence warns that consensus building can be challenging [27]. Furthermore, structural challenges that communities identify as targets for change, such as poverty and political instability, have created obstacles to problem-solving leading to frustration and barriers to community empowerment [26]. However, these issues can be overcome, as seen in a recent application of CC in Zambia for the improvement of women and child health outcomes, where a range of community-led activities responded to structural drivers of poor outcomes, including the construction of new health centres, alongside typical health information programmes [29].

Given the capacity for CCs to illuminate a wide range of concerns linked to health challenges, while providing a pathway to meaningful dialogue between communities and researchers, we felt the methodology could provide a more systematic approach to community engagement in the design of our intervention. We describe our approach, as well as findings generated from its use in subsequent sections.

### The INSPIRING intervention: a whole system approach to tackling infectious diseases in children under five

Formative research was carried out as part of a larger INSPIRING programme in Jigawa State Nigeria, which uses a cluster RCT approach to impact evaluation [30]. The project involves multiple partners and was designed through an iterative co-design process with the funder, implementer, evaluators, government and local community members. The intervention is a locally adapted ‘whole systems strengthening’ package of three evidence-based activities: community Participatory Learning and Action (PLA) groups; Partnership Defined Quality Scorecard (PDQS); training and engagement between caregivers and health facility staff, mentorship and provision of basic essential equipment for child health. The inclusion of community members in the co-design process was achieved through an adapted CC approach, which allowed us to confirm the details and implementation/delivery strategy of PLA and PDQS community link intervention.

#### Research setting

Research was completed from November 2019 to March 2020. Jigawa is one of Nigeria’s 36 States, with an estimated population of 4.3 million constituting mainly Hausa-Fulani tribes, and a small proportion of Manga and Badawa, tribes [31]. People live in extended family compounds typically comprising of two to five households with children, parents, grandparents and other siblings. 98.9% of the population practices Islam, while 1.1% are indigenous Christians. Agriculture is the occupation for 80% of the population and most live in rural areas. Two-thirds of the population (69%) live in severe poverty, with 50.3% belonging to Nigeria’s lowest wealth quintile [31].

#### Implementing community conversations for formative co-design research at scale: a new method

To ensure meaningful involvement of the community in shaping the intervention design, RAB developed an adaptation of the Mandela Foundation CC approach, to engage with community members at scale. Though typically applied as an intervention itself, our reformulation was designed to deepen engagement with the communities of men, women and health practitioners who will engage with the proposed interventions. Conversations were structured in a similar manner to the PLA group component of the intervention, providing an additional layer of depth for understanding the acceptability of the approach in this context. Our adaptation included four of the five CC stages: Relationship building, Concern identification, Concern Exploration and Reflection and Review. Our decision to exclude the *decision-making* stage was linked to the ability for this stage to turn the approach into an intervention itself, as it is oriented to deciding and acting on how to answer the problems that have been prioritised in previous stages. To run this stage would have comprised the ability to establish true baseline prior to the trial intervention being delivered in later months. Interactive CC discussions explored: perceptions of key concepts underpinning the proposed interventions (e.g., challenges related to child health); relationships with health care workers; and how key intervention components would work best in their communities, including location, timing for delivery of groups and incentives.

#### Sampling

Three categories of Jigawa community members were invited to participate in CCs: men, women and healthcare workers. Participants were recruited using a blend of convenience and purposive sampling. The inclusion criteria included any adults who were parents and caregivers of children under five, aged 18-49 years, or health care staff. To identify eligible candidates, the research team walked around the communities with a village gatekeeper someone with intimate knowledge of the geographical and social community, who is well respected and known by those living in the area. This resulted in a combined population-level availability and targeted sampling. Consent was obtained from participants who were then invited to gather at the meeting point.

During CC meetings, participants were broken down into sub-groups. This ensured that any participants with have limited agency in mixed settings (such as younger women or lower ranking wives, which we observed in this community in earlier work (see [32]) had opportunities to contribute ideas and shape discussions and at each round. Older women (age 31–49), younger women (age 18–30), and men (age 18 above) were placed in groups. Given that men with children in community settings were found to have so much power in our scoping research [32], and based on the perspectives of research team members who are local to this context (FS, IH, AB, ) we did not anticipate the need to separate men into sub-groups by age. Because of the number of participants at each site, we had groups running in parallel on each day, with a maximum number of 10 participants per sub-group working together at a time.

#### Procedure

CCs were conducted across 2–3 days, per ward. Wards were selected based on consultation with the INSPIRING team, to ensure a balance between rural and peri-urban sites, as well as moving across the whole local government area. Conversations were held at primary school sites, which was agreed upon following earlier consultations with community gatekeepers.

Each CC session started with the researcher welcoming participants and recording their socio-demographic characteristics. Facilitators provided a brief introduction in which they explained the project, its partners, aims of the conversations, and established ground rules for the conversations. Facilitators were responsible for guiding the conversations with open-ended questions and activities, which are detailed in the Supplementary materials Discussions in sub-groups were not recorded, but recordings were taken during the larger discussions where each group reported back to the full group. Note-takers captured additional reflections from sub-group and large-group discussions.

We ensured the process was not dominated by any single voices or groups, by giving all groups the opportunity to participate and by distributing tasks within the smaller groups, i.e. some volunteered to draw, while others generated ideas of which they all deliberated on. Refreshments and transportation reimbursements were given to participants at the end of each session (Table 1).

**Table 1:** Overview of procedures in CC adaptation process

Sessions	CC stage	Activity conducted	Time for activity	# of facilitators required	Resources required
Session 1	Relationship building and concern identification	Body mapping and discussion	1.5 h	2 minimum	Flip chart paper Large paper cut-outs in shape of a body Coloured marker pens Tape recorder
	Concern exploration	Venn diagram and discussion	1.5 h		
Session 2	Concern exploration	Venn diagram and discussion (if not done in session 1)	1–2 h	1-2	Flip chart paper Coloured marker pens Tape recorder
		Community mapping and walking map	2 h	2	
Session 3: Concept testing and meetings with additional wards	Reflection and review	Focus group discussion	2–3 h	2	Tape recorder Flip chart paper

#### Participatory activities

**Fig. 1:**
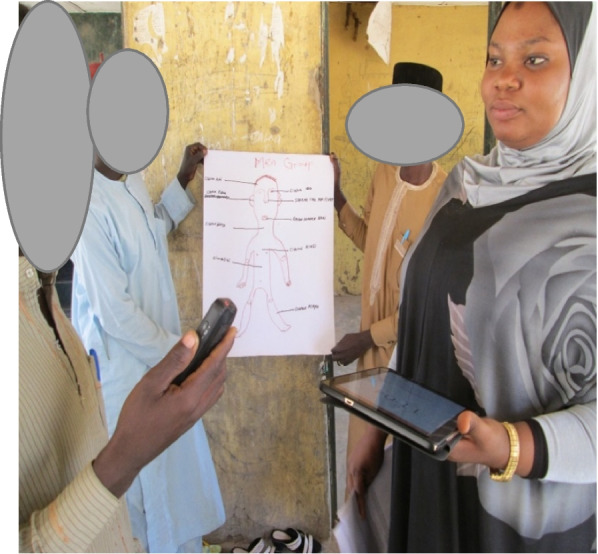
Men’s group: showing body map of a child with associated risks factors

**Fig. 2:**
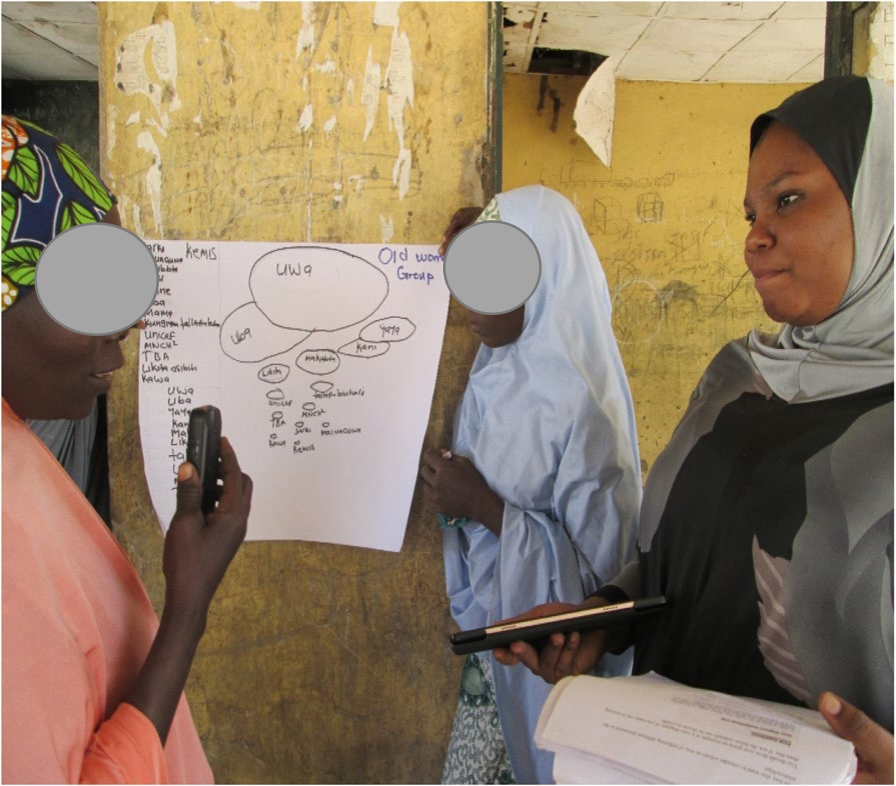
Older women’s group — Venn diagram of Community power dynamics

Participatory learning activities [33] provided opportunities for people to share and contribute to debates, without direct questioning, and created a level playing field among participants with limited formal education — Figs. 1 and 2 demonstrate some of these activities.

### Localising risks to child health: body mapping

Body mapping activities are widely used in research studies that seek to understand how participants make sense of bodied manifestations of pain, emotions, and illness, using illustrations [34]. We used the approach here to make discussions about risks facing children more tangible, allowing participants to anchor discussions to maps of an imagined child. Our approach to facilitating this activity is described in supplementary materials document 1.

### Understanding relationships: stakeholder and power mapping (Venn diagram)

A key part of intervention development is the process of stakeholder mapping. We expanded stakeholder mapping, by including a Venn diagram activity (see Fig. 2). Using these methods, allowed us to explore not only stakeholders, but to tangible represent the relationships between different actors. This is particularly important given existing understandings of gendered power dynamics in the region [3].

### Understanding space and place in relation to child health — community mapping

**Fig. 3:**
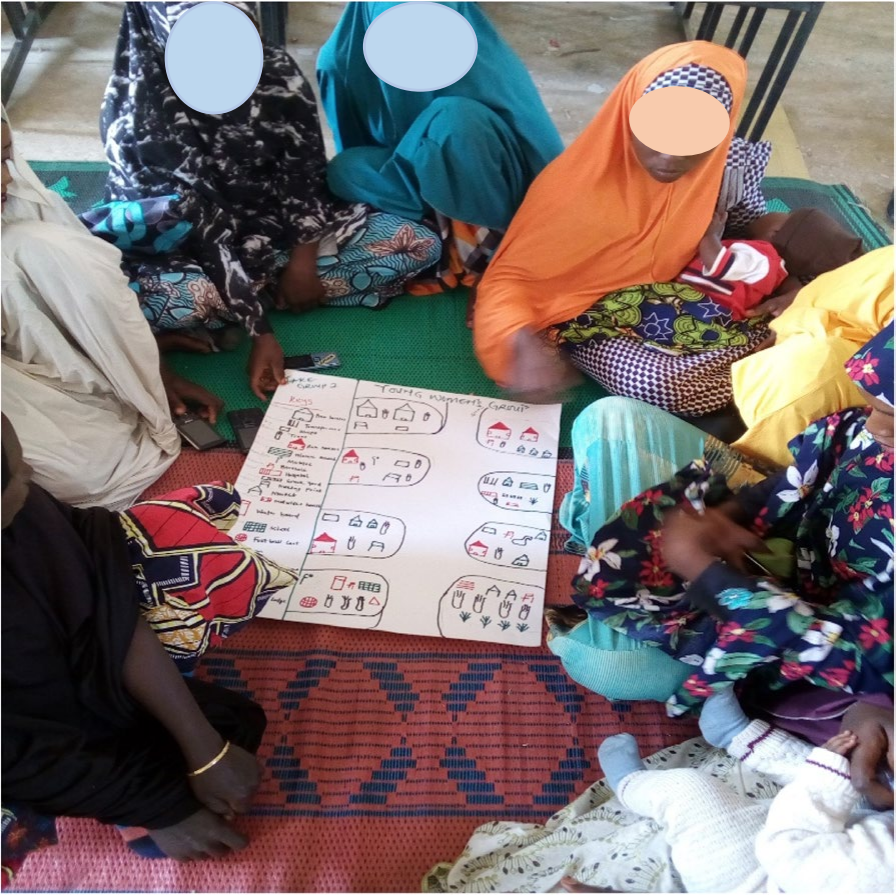
Young women’s group community mapping activities

Community-based and community-led interventions demand an understanding of community that is as complex as possible, though this is rarely the case. Typically, the emphasis on community stops at the geographical, with concerns about distributions of illness or demographics. In our context, we sought to explore community as a more complex entity, acknowledging three often under-appreciated dynamics related to communities of place: (1) social identity processes (how people identify and relate to each other) linked to the use of space; (2) community of practice and action — created through engagement in shared activities; and (3) symbolic communities — linked to cultural sub-groups. The mapping activity (see [35]) began with a process of identifying landmarks and resources within the space but expanded beyond this to enquire about how different groups within a community linked to the above dimensions make use of this space. Details on how this activity was carried out are included in supplementary data document 1 (Fig. 3).

### Focus group discussions: reflection and review through concept testing

The final stage in the community conversation process involved dialogue-based concept testing [34]. This allowed us to share findings from the community conversations with additional community members. Broadly speaking, our efforts were in line with member-checking approaches in qualitative research, which increases the trustworthiness of data and enables researchers to ensure that participant meanings are not supplanted by researcher priorities and aims for knowledge production [34].

We presented synthesised data and a proposed intervention structure to participants in two types of focus group settings: (1) discussions with sets of new participants in the six wards we had been working in previously (*n*=54), and (2) discussions with participants in the 5 governmental wards where we had yet to collect data (*n*=45). This member-checking approach fits with the wider epistemological purpose of community conversations, where the goal is to remain rooted in community-owned knowledge production. However, by also including new participants, our emphasis extended beyond validation, allowing us to explore whether the perceptions and views of other community members resonated with others, to identify dissonant voices, and create opportunities to add new data or perspectives to our findings before rolling out the intervention [34, 36]. This adaptation is particularly important for trial-related work, where the approach will eventually be rolled out to large portions of a population. Details on activities are described in supplementary data file 1.

#### Data collection, processing and analysis

Three local research assistants (RAs) were recruited and trained by FS, who is local to our study community. In addition to exploring study objectives and sampling processes, role play was used to introduce RAs to community conversation procedures. This allowed us to confirm the resonance of the activities with people from this community and refine our approach if needed. The standard operating procedures for the method were provided and included sample probes to expand on responses in sessions. All conversations and interviews were recorded, transcribed verbatim and then translated into English by the local RAs.

Data analysis was completed using a codebook approach to thematic analysis [37]. Following an initial reading of a selection of transcripts by the lead author RAB, a preliminary codebook was created, to structure a focused reading of data, to provide answers to specific questions in relation to intervention development. This was then used by additional members of the analysis team (FS, AI, AAB), who expanded on these initial structures individually. Individual updated codebooks created by other analysis team members were consolidated by RAB, looking for convergence/divergence across themes. A final codebook was circulated to the analysis team, and additional members of the study team (TC) for agreement on the summary analysis at the preliminary stage.

## Results

A total of 320 participants from six wards were engaged in the conversations, with an average of 53 participants per ward (Table 2). Below we present findings from our analysis that were used to finalise the content and structure of the PLA intervention. These findings are linked to the codebook, which we provide in the Supplementary materials. We report specifically on.

**Table 2:** Distribution of participants per sub-group for CC and focus group discussions (FGD), in the first six wards

Wards	Number of CC participants/wards	Number of CC participants/sub-group	Number of FGD participants/ward	Number of FGD participants/sub-groups
Fake	54	Older women:19 Younger women:16 Men: 19	30	Older women: 5 Younger women: 5 Men: 5
Kiyawa	52	Older women:16 Young women:19 Men: 17	30	Older women: 5 Younger women: 5 Men: 5
Andaza	54	Older women: 18 Young women: 18 Men: 18	30	Older women: 5 Younger women: 5 Men: 5
Balago	54	Older women: 18 Young women: 18 Men: 18	30	Older women: 5 Younger women: 5 Men: 5
Shuwarin	52	Older women: 18 Yound women: 18 Men: 16	30	Older women: 5 Younger women: 5 Men: 5
Katanga	54	Older women: 17 Young women: 17 Men: 20	30	Older women: 5 Younger women: 5 Men: 5
Total	320			

### Uncovering hidden processes that shape the impact of interventions

#### It’s a man’s world, but a woman’s decision? Power dynamics between stakeholders in communities

In developing an understanding of power, we identified that gendered power relations shaped the use of space and ownership over decision-making linked to child health.

There was a lack of consensus about who held the most power in relation to child health. Women’s groups identified women as having more power, and men identified men as having more power. Our work identified the existence of a decision-making pathway, where women and men’s power worked in parallel ways, through different forms of power, to achieve particular ends. For example, men’s influence was associated to their economic power, as they have primary decision-making in households as is common in patriarchal societies. [38]. Women’s power reflected what scholars would label as symbolic power [39, 40]) — created through an intimate knowledge of a child’s life, needs and responsibilities. This was felt to only be held by women. Because of this, women asserted that men were only brought into decision-making processes, if all other outlets for response had failed, or financial resources were required. Older and younger women’s groups across all six wards agreed on this division of labour.

[The] mother is responsible for taking care of the child, to the extent of doing things that even the father cannot do for him (child), even if the father does not have money, she has to find means of taking care of him, it is only when she does not have that the father will provide – she (the mother) takes care of the child more than the father. – Participant from Young women’s sub-group

Other stakeholder groups such as neighbours and other blood relations (such as grandparents and siblings) were described as alternative sources of financial resources — highlighting that the community is built on important webs of financial capital that could potentially shape how people access information and make decisions. For example, in one ward, participants indicated that ‘their doctors’ (how community members described any health staff at local clinics) were not as powerful in terms of child health as neighbours — because in a health crisis, they would go to them first for information and support.

Neighbours form important kin networks, provide support, advice and access to resources that a family member may not have, or if a husband is away, then they could also provide. These networks are key for the mobilisation of various forms of resources and become opportunities to overcome products of structural forms of power (such as the organisation of state structures and their management).

Discussions about the role and presence of external groups and agencies illuminated that despite an acceptance of the important things that organisations like UNICEF, Save the Children International and USAID do for child health, they couldn’t make specific reference to the actions they contributed, or the motivations for their engagement. As noted by a participant from the men’s group “…they [UNICEF] do things we don’t know how to do – [but] We can’t say where this organisation came from.” This is particularly interesting, as there is a recognition of power and capacities of these external actors, which does not automatically translate to an understanding of motivations or purpose behind their involvement. This raises concerns about how trust can be established between community members and external actors, as evidence from other settings suggests that in research among marginalised groups, trust can be limited when groups have little to no understanding of service aims and priorities 41). In some wards, participants acknowledged the failings of political power and process — noting explicit reasons for not including political figures as key players in determining well-being. In two wards, there were discussions of the inability for politicians, specifically at the local government level to be trusted. As highlighted in the below excerpt from conversations in one ward:

I: can you tell me the reason they [politicians] are not important?

MG participant: because as a politician, you can never get what you want from him at that time – whenever you are in need, he will keep promising you… anything that has to do with health needs instant help.

OWG participant: … they don’t help their fellow men, even on child health. It’s even rare of we the females to go to them for help when we are taking our child to the hospital.

YWG participant: …why we say they are important is they [set] programmes, like polio vaccination, and in some areas the health workers listen to directives from the politicians.

MG participant: …. But to be honest, we have not seen in any way that they are important to improving child health because the only [ones] that have a say in our community are community leaders (heads) or village heads – politicians only know how to deceive…

MG participant: … if you want people to come together a politician will never bring [them] together for you… but the village head or the community leader will organise people for you because we all respect them and work with their instruction.

Relationships between actors involved both vertical and horizontal communication and flows of power, which hindered our ability to clearly map flows of information. For example, participant accounts indicate that the district head and ward/community heads largely disseminate information down towards communities. While this cannot be orchestrated without the approval of the Emir, a local leader who is responsible for large geographical locations, it reflects that power can also be linked to expertise, and as a result in some contexts, the King’s power can be deferred to Ward and community heads. For example, an Emir may not be involved in everyday decisions that are community specific. District Heads have the liberty to make certain decisions, but with limitations. In other instances, village/community or ward heads who make local decisions may defer to someone younger or who has more expertise in certain areas. For example, in one of the concept testing wards, when our research team organised a meeting to explain the project aims, a community head identified himself as ‘too old to understand’ and referred the team to a younger community head to organize the actions of the team. Furthermore, not all wards reported the presence of a ward head — though community heads were unanimously referenced by all groups. In most cases, dissemination of information/innovation begins with the district head and moves downward throughout the community. Everyday citizens seemed to have the least opportunity to generate information, with women the least likely to be seen as a source of knowledge outside of child-related dynamics.

Given evidence which suggests that an underappreciation of power relationships can negatively impact community health interventions [41], two actions were taken. The first was the addition of a module within the PLA intervention manual on family relationships and power in the household. The second was to ensure that PLA groups were facilitated with women and men separated initially, then bringing them together for specific planning meetings in preparation for reporting activities to the community link aspects of the intervention. These approaches have been applied in other settings where patriarchal or traditional structures have influenced the participation of certain actors within an intervention [42, 43].

### Where can we meet and who will speak? Problematizing the use of space

#### Gendered use of space

Participants from all wards agreed that there were clear gendered rules about the use of space in communities. Specific sites such as graveyards, Mosques and the market were labelled as off-limits to married women, by all participants, regardless of the presence or absence of a chaperone. Men were prevented from entering any space where women met in groups, or from entering households where women were present in the absence of the male head of household.

to be honest, going by Islamic rules it is forbidden for women to go to the market, so we are only abiding by such rules, they don’t go to the market at all (Participant from Men’s group 2)

men are not allowed to go to women’s gathering like naming ceremony or wedding (participant from Young Women’s group 2)

However, all groups also noted that there were situations where women’s mobility became more flexible. For example, with their husband’s permission women were allowed to leave their houses without the presence of a male chaperone, if it related to taking action on health-related matters — emphasising women’s participation in health decision-making described earlier. The district head would give permission for men and women to be together in the absence of their husbands. Information about these decisions is passed down from district heads to husbands and male heads of compounds. In light of this, plans for the community link intervention where men’s and women’s groups were required to meet and establish shared plans and priorities to be considered at health forums, could only continue with explicit permissions for these meetings from the district head.

#### Marginalised groups and use of space

All groups highlighted two particular communities — groups that represent the most marginalised and this marginalisation shapes community use of space. Fulani people are traditionally cattle farmers — this means that they are fully engaged in farming activities (including raising livestock) and live on the margins to help protect the wider community from damage or illness caused by exposure to livestock, and for easy access to grasses for their cattle. This was noted by participants as an active choice among farmers — not forced through cultural norms. All wards noted that there are certain times of the year when Fulani people come into town, to sell their products, and during these times use space equally alongside others in the community.

It’s only the Older Women’s group 1)

most of us (Fulani man attested) stay near the bushes and farm lands because of the animals we rear, so that they cannot easily go into the community and destroy people’s things (Participant from Men’s group 2)

Small numbers of Hausa tribe members also live on the outskirts of communities. They are often very poor, and cannot afford land of their own, or building materials. As such, land is often gifted to them by the district head, but this land is on the outskirts of the community, leading to experiences of marginalisation.

Children were described as able to access any community spaces, as long as their safety/health was not placed at risk. For example, children were not allowed to be in places where they could be exposed to Jin (described as spiritual problems and possession that could lead to paralysis of hands/legs), including dirty places, graveyards, wells, or main roads.

like the traditional event men organized, children are not allowed to go there, children are not allowed to be seen at the main road, and also small children do not go to the graveyard and also playing close to river (Participant from Men’s group 2)

place like the grave yard, children are not allowed to play there, and also main road to avoid accident (Participant from Young women’s group 1)

### Participation does not cost a thing, but it helps: reflections of systems of compensation for community interventions

Evidence around participation increasingly critiques the ways in which the time and contributions of communities are reimbursed [44]. Our member-checking approach allowed us to identify a tension around this particular aspect. In the six wards where full CC activities were carried out, financial compensation was identified as important.

The group members should be given fund or capital to start up a business, with that they will know that an organization came not only to take care of the children but to also empower the parents (Participant Male subgroup6 )

you know we people like to be given money in anything, like on your first visit here you asked of our names, contacts and business, so we feel if you can empower us to strengthen our business we would be very happy (Participant, Male subgroup 5 )

However, in non-CC wards, monitoring from the overseeing organisation (mentorship, praise and check-ups on progress) was named as a form of compensation to consider. Several types of compensation were proposed: book and pen, training, t-shirts or uniforms, and certificates.

All wards noted that decisions linked to child health were enabled or hindered by financial resources, which resonated with earlier scoping research conducted in 2019 by our team [32]. This informed our team decision to have an approach to compensation, that contributed useful materials to a household (i.e., baby carriers for women) and allowing groups to decide on the timing and locations of their meetings throughout the study. Groups were advised to select times that did not put earning potential at risk and were flexible around seasonal shifts (i.e. rainy seasons). As groups have now begun to meet as part of the trial, we can report that this openness resulted in nearly all groups meeting on Saturdays, and men’s groups have shifted timing in response to the onset of rainy seasons. We anticipated that it would be likely that many PLA group activities may lean towards income generation in study settings, and as such we prepared facilitators to have advice for these processes if needed, with guidance and examples of different types of income-generating activities that have been used in other settings.

## Discussion

The application of community conversations within formative research enabled us to gather deep and rich understandings of communities, contexts and power dynamics, prior to finalising the design and implementation of our intervention. Our approach in this study avoids the assumption that generating an understanding of communities of place — even within a singular state, is sufficient to understand and define an approach that 1 day hopes to run across an entire state. By working across the local government area, and importantly, dividing participants into smaller groups that represent key stakeholders for an intervention, we were able to confirm a series of priorities and perspectives that could be shared by the whole group, increasing the likelihood of uptake when the formal intervention was rolled out in the future, because of a deep understanding of the varied contexts and experiences of these contexts among participants.

Participants saw this procedure as an opportunity to learn, unlearn and relearn. In all conversations, participants appreciated the fact that they were able to learn new things from other members, they embraced the fact that they could argue out points and reach a concensus of what is obtainable/workable within their community, citing examples from programmes that were carried out in the past and how they could be leveraged on.

While there is much said about the importance of participation in trial development, the type and aim of participation matters. As mentioned earlier, Susan White’s paradigm [24] is anchored in critiques of participation in development settings, namely the ability of power to shape the possibility for participatory approaches to be successful. Nominal participation and instrumental participation, are two forms of participation where the aim and purpose is to serve the purposes of practitioners and formal institutions. In these cases, participation functions as a route for inclusion, but community actors have very little decision-making power within the wider project processes. These types of participation are most common within trials, and typically particular feature in typical formative research studies using standard qualitative methods.

In response to critiques in wider development spaces, we have been called to prioritise opportunities for more representative and transformative forms of participation. While representative forms of participation are interested in both inclusion and sustainability within projects, transformative participation views the inclusion of community actors as both the means and the end, such that community involvement results in opportunities for meaningful change, that has an impact on both processes and outcome [24].

Our aim was to create opportunities for representative and transformative types of participation within the trial process, with an emphasis on intervention aspects, among a large group of community members in the sites where our trial would eventually be implemented. CCs offered the opportunity for community members to fully shape the design of the intervention and incentive structures. This, alongside wider processes of collaboration and consensus to shape some aspects of design, contributed to representative forms of participation. Because final decision-making around the intervention remained with the wider team, transformative participation was not achieved in this instance. However, as outlined in the results, learnings from the CCs also shaped the structure and content of the PLA intervention, the mode of delivery, as well as aspects of evaluation [45].

We also believe that through dialogue coordinated in a respectful and open forum, the approach established opportunities for interpersonal transformation, with young women able to feel heard and have their views acknowledged in forums where this is often unlikely. Key to this, was the use of visual and image-based activities — as these ensured that even those with low literacy were not excluded from dialogue opportunities. Thus, the activities not only gave members a sense of ownership over the intervention, but had the opportunity to improve future communication within community networks more broadly.

Furthermore, embedding CC methodology within trial design processes provides a pathway to engage everyday citizens as experts in the processes of knowledge production in more meaningful ways. Recent work by Abimbola [7] articulates the importance of exploring four units or sites for knowledge production — two of which are often underappreciated in the global health landscape: Primary users who use knowledge to transform their own health (everyday emancipators) and those who use knowledge for change (implementers/plumbers). While Abimbola speaks of activists and NGO staff as primarily embodying these spaces (respectively), in our approach, we articulate pathways to view everyday citizens engaged in projects of survival as the emancipators that they are actors who negotiate, have intimate knowledge of their daily efforts to transform their own lives, and what ‘successful’ interventions could and should look like in order to support their efforts. Methods such as ours are critical if we are to resist the ways that everyday citizens are often excluded or silenced within science [46].

### Limitations

Despite the strengths of CC approach as we have used it in trial design, some challenges remained. For example, in Nigeria, large gatherings of people can be mistaken for/perceived as a call for political aims. In implementing this methodology, the purpose should be well communicated not only to study participants, but also to the broader community as well. While participants were fully aware of the purpose of the conversations, other members of the community who had not heard about the meeting, initially felt that it was a political gathering and questioned why they were excluded, and they tried to politicise it. In this instance, we were able to reschedule our meetings for another day, ensuring a wider range of invitations. This suggests that for others applying this approach in highly politicised contexts, CCs should be opened even wider, more in line with their original approach with open public forums.

Our sampling approach was designed to include anyone eligible for the final intervention, ensuring that participants would also be likely to include in the trial at some later stage. By organising activities within this approach by sub-group, we attempted to reduce the potential for exclusion faced by some community members (such as young women). However, this did not allow us to include all vulnerable groups — as some sub-communities (such as the poorest members of the Hausa tribe) may have struggled to participate over the multiple days of data collection. Unfortunately, we did not record details on non-attendees on the second day of CC sessions, which is another limitation. Future studies may want to explore how actors ‘voting with their feet’ promote additional learnings about the potential interventions.

The need for repeat attendance at CCs can influence the effectiveness of the approach. To counter this, we held our conversations over the course of 2 days to reduce demands on time. in contrast with the original application, which can be run over weeks and months. In applying CCs within trial settings we suggest an acknowledgement of how time away from employment, school or training can impact well-being as well as the ability to engage in these processes. Appropriate compensation schedules that align with the time investment of communities are critical and are an equitable way to acknowledge the importance of everyday knowledge to scientific processes especially when this method extends for longer periods.

Finally, the approach is also resource intensive. Our ability to conduct this approach effectively was facilitated through the involvement of four community researchers devoted to the qualitative aspects of the formative research. It is imperative that these aspects of research are appropriately costed, to enable in-depth exploration.

## Conclusions

Our work shows the value of using a CC methodology to inform the intervention design and delivery within a trial. Working within the confines of a pragmatic trial, we sought opportunities to ensure that everyday citizens s had more involvement in the early stages of our work, beyond the standard practices seen in literature. To our knowledge, this is the first use of CCs in trial intervention design and delivery. Furthermore, we believe qualitative scoping research of this scale is very rare. . Though not all stages of the trial process were influenced by data collected by CCs, our experience highlights that it is a feasible approach to gaining an in-depth understanding of community perspectives, contexts and intervention feasibility in a way that extends beyond current formative research approaches. Importantly, it shows a route to widening participation to include everyday citizens in the development of science that seeks to be for their benefit.

## Supplementary Information

**Additional file 1:** **Supplementary material.** Detailed methods for participatory activities used in community conversations sessions. **Supplementary materials appendix 1.** Concept testing guide for community link: VDC discussions. **Supplementary materials Appendix 2.** Concept testing for community link: PHC interviews. **Supplementary materials 2.** Summary descriptions of stakeholders in child health as defined within CC discussions.

## Data Availability

All data required to understand our analysis are provided in supplementary data tables. Full focus group transcriptions may be available for secondary analysis, with the permission of the wider consortium.
