# Supplementary material for: Whose knowledge counts? Involving communities in intervention and trial design using community conversations
Source: Trials. 2023 Jun 7;24:385. doi: 10.1186/s13063-023-07320-1 (PMC10249250; doi:10.1186/s13063-023-07320-1)
Supplement: Supplementary file 1 — Additional file 1: Supplementary material. Detailed methods for participatory activities used in community conversations sessions. Supplementary materials appendix 1. Concept testing guide for community link: VDC discussions. Supplementary materials Appendix 2. Concept testing for community link: PHC interviews. Supplementary materials 2. Summary descriptions of stakeholders in child health as defined within CC discussions. [file 13063_2023_7320_MOESM1_ESM.docx]

Supplementary material:

**Detailed methods for participatory activities used in community conversations sessions**

**Activity 1: Body Mapping**

*Start:*

Prior to starting the activity, Participants were shown samples of body maps drawn by the group facilitators during training. These maps included a full human body with arrows directed at body parts with perceived health issues and concerns depicted in images (Fig. 2). Facilitators also explained the meaning behind labels and arrows.

*Activity:* Divide participants into their appropriate sub-group (men; young women, older women). Paper, markers, pencils and erasers were given to each group and they were asked to create with body maps to explain perceived health issues relating to children in their communities. Facilitators visited each group, providing assistance as needed. Participants were advised to write in the language they were most comfortable with, or use images instead of words if they preferred.

After 45minutes -1 hour, representatives from each subgroup were invited to present their body maps to the whole group. Researchers facilitated discussions to explore similarities and differences between groups. Finally, researchers worked with group members to create priorities around three broad areas of risk facing children’s health: environmental, spiritual and emotional risks.

**Activity 2: Stakeholder mapping and power dynamics in communities**

*Start*: The aim of this activity is to get information on stakeholders at households, community, government, and NGOs levels to engage during intervention design on children’s health related matters and how much influence they have on them.

*Activity* This technique was simply a collection of circles, with each circle representing a different individual/group or organization. the size of each circles reflects the relative importance of the group represented-the smaller the circle, the less influential the group. This activity provides information on groups of people and institutional structures in communities that are important to include in plans and efforts to improve children’s health. Groups were asked to generate lists of stakeholders related to child health, then re-arranged actors in order of importance or priority to child health (Fig 3). Thereafter, they proceeded to draw circles to represent the groups of stakeholders, using size of circles to represent how much power or influence the group had on decision making. In situation where there was intersection of roles between stakeholders, there were overlap of circles.

**Activity 3: Community mapping and transect walks.**

*Start:* Community mapping was conducted to identify important landmarks in the community. The aim of this method was to have an idea of resources available within communities and how to leverage on them in solving their problems.

*Activity:* A skeleton map of a fictional geographical community was shown to participants as an example. They were then asked to draw their own community, indicating the different asked to draw their own community with important places and resources distinct to their communities (Fig 4). After they had drawn their maps, these were compared and shared across sub-groups and important features were highlighted, this includes meeting places, Market square, Major Mosque (where Friday prayers are done), traditional leaders house, shops etc. Two participants from each group volunteered to walk facilitators around their community while they showed and explained all landmarks, they had on their drawn maps (i.e., a walking map). They were required to answer specific questions about the use of space, specifically – the who, where why and how of the use of space and resources.

**Final sessions: Concept testing**

Procedure: Focus groups were organised with 7-10 participants (see Appendix 1and 2 for questions). FGDs should present participants with the main themes from the initial analysis of community conversations. In particular: lists of actors identified as primary stakeholders of concern in child health settings, barriers to health care. FGDs should be led by two researchers, a facilitator and an observer took notes, and take no longer than two hours.

Given that our aim was to explore the nature of the intervention, we also used this opportunity to engage staff from PHC centres to explore their perspectives of the community link aspect of the intervention. Health care providers interviews explored topics on quality health care, challenges encountered while delivering quality health care, relationships between staff and community members. These data are described elsewhere (See Illuiano et al., forthcoming).

**Supplementary materials appendix 1: Concept testing guide for community link: VDC discussions**

**Background questions**

1. How long has this [VDC group] been active?
2. What is the main aim of your work together in this community?
3. What are your thoughts on the biggest health challenges facing children in this area?

Please prompt for…

- *how do you think these problems can be solved?*
- *What role do health services and staff play in these solutions?*

**Specific questions about community link intervention**

1. How would you define ‘quality health care’?
   1. How would you define ‘quality care’ for children?
2. What is the biggest challenge to the delivery of good health care services in this area?
   1. *From your perspective*
3. Do you think that health service staff care about quality of services for children?
   1. If yes - Why do you think so? Can you give an example of a time where you felt this was true?
   2. In general?
4. How have you worked in the past with health service and the health system to improve health in the community?
   1. *If they have never done this – why have you not worked with health services before?*
   2. *If they have – what has been good about it? What has been challenging?*
5. How do you think the wider community feels about working with health facilities and staff?
6. Is there trust between health facility staff and communities?
   1. *If yes – can you give an example of a situation that highlights trust?*
   2. *if no – why not? Can you give an example of what makes you feel this way?*
7. How do you think you could build more trust between communities and health services?
8. Do you think that communities and health services can work together to improve services?
   1. *If yes – What reasons would communities and health facilities work together? Can you give an example of when this has happened before?*
   2. *If no – why not? Can you give an example of what has made you feel this way?*
9. How do you think that communities can contribute to improving health services?
10. If you were to try to make relationships better between communities and health services, what would you do?
    1. *Would you hold meetings? Public events?*
    2. *What should these meetings look like?*
    3. *How often would you need to engage with health services to make things better?*

**Supplementary materials Appendix 2: Concept testing for community link: PHC interviews**

**Background questions**

1. What is your name and how long have you been at this PHC?
2. What are your thoughts on the biggest health challenges facing children in this area?

Please prompt for…

- *how do you think these problems can be solved?*
- *What role do community members play in these solutions?*

**Specific questions about community link intervention**

1. How would you define ‘quality health care’?
2. How would you define ‘quality care’ for children?
3. What is the biggest challenge to the delivery of good health care services in this area?

Please prompt for:

- 1. *From your perspective*
  2. *From the perspective of your staff? (i.e., PHC nurses etc.)*

1. What barriers do you face in trying to deliver good health services in this area?
   1. Prompt specifically for children’s health services
2. Is anyone in this area doing work around improving quality of services
   1. In general?
   2. For children?

Please prompt for:

- *is there a health facility quality improvement committee?*
- *Is there a VDC? /VHC? Have you worked with them before?*

1. How do you think the wider community can contribute to improving quality of health services?
2. How do you think the wider community feels about working with health facilities and staff?

Prompt for

- Is there trust between health facility staff and communities?
  - If yes – can you give an example of a situation that highlights trust?
  - if no – why not? Can you give an example of what makes you feel this way?
- How do you think you could build more trust between communities and health services?

1. What reasons would communities and health facilities work together? Can you give an example of when this has happened?
2. How do you think that communities can contribute to improving health services?
3. If you were to organise meetings with communities to make positive changes around things we’ve discussed:
   1. What should these meetings look like?
   2. How often would that need to happen to make your engagement productive?

Supplementary materials 2: **Summary descriptions of stakeholders in child health as defined within CC discussions**

|  | Stakeholder | Role in relation to household and child health |
| --- | --- | --- |
| 1. | Mother | Back bone of family and children - closer to them than any other person, primary responsibility to ensure child’s health and wellbeing.  “before a father will do something for a child, once, the mother might have done this like 20 times – our religion recognizes a mother before a father” – CC participant, Older woman’s group, Balago Ward |
| 2. | Father | Breadwinner of the family pays bills, gives orders, makes decisions, and can help with health and wellbeing. |
| 3. | Grandparents (Grandmother named most often) | Take on the role of parents, in the absence of mother/father. Play the role of parents, but also will contribute to solve problems with health. |
| 4. | Uncles | Act as father on child health issues in the absence of father – they can take care of the young ones. |
| 5. | Siblings’ (Elder brothers and sisters) | Assist (financially) parents in taking care of their younger ones |
| 6. | Neighbors | Help each other when there is problem or when the father is not around to pay bills |
| 7. | Friend | Gives financial support and advice when children are sick |
| 8. | Schools | Schools ensure they have first aid which they administer to children before taking them to the hospital |
| 9. | Teachers | Give first aid treatment to children when they come up with illnesses while they are in school |
| 10. | District head | Ensures enforcement of traditional orders/laws/directives and also serve as a gate keeper. Responsible for multiple kings within a district. |
| 11. | King | A King per ward and is a gatekeeper for access. Receives first hand of information on whatever comes into the community and others must work with king’s permission to deliver interventions. |
| 12. | Ward head | One head per ward, but not noted in every ward that completed the CC methodology. Role is similar to district head, but with a closer proximity to communities. |
| 13. | Community head | Multiple communities. Each gives directives, owns the power and will of his community, equips health facility – can contribute funding for poorer families if needed |
| 14. | Village head | Aids the dissemination of information to the grassroots (everyday men and women) works with committees within community, and also work with community head directives - links back and forth between grassroots and higher levels of structure/decision making (literature discusses this role as brokerage) |
| 15. | Imam (Religious leader) | Preaches, christens a child, helps to pass information and offers prayers and healing |
| 15. | Religious teachers | Offer prayers in situation where the root cause of an illness is unknown to parents |
| 16. | Governor | Equips facilities with drugs |
| 17. | Counselor | Fights for the wellbeing of the community to ensure facilities are equipped with drugs |
| 18. | Chairman | Solicits/raise fund for health-related issues |
| 19. | Vice chairman | Plays the role of the chairman if he is not available |
| 20. | Hospital | A place where patients get appropriate treatment |
| 21. | PHC head (in charge) | Runs the total welfare of the PHC center** |
| 22. | PPMV | Drugs which are not available at the hospital are bought there |
| 23. | Health care personnel | Go to the community to check on members welfare |
| 24. | Doctor | Diagnose, treat and prescribe drugs to patients. Many other stakeholders were listed as more important, as you were often engaging with them first (i.e. neighbours, other family members). |
| 25. | UNICEF | Gives free drugs and plumpy nut (Tamowa) to malnourished children  *“UNICEF gives more contributions to the improvement of our child’s health than Ward heads – they [UNICEF] do things we don’t know how to do. We can’t say where this organisation came from – but our ward head’s - we know where they come from”* CC participant, Men’s group, |
| 26. | WHO | Gives free drugs |
| 27. | WINNN | Take care of malnourished children i.e., give plumpy nut |
| 28. | SCI | Also contribute to child health. **Note: they were not specific about their role** |
| 29 | USAID | Contributes to things related to child health. **Note: they were note specific about their contribution** |
| 30. | ACF |  |
| 31. | WASHCOM | Brings improvement to the community I.e., ensures proper sanitation |
| 32. | JISEPER | Help in managing generated wastes |
| 33. | RUFT |  |
| 34. | Volunteer community mobilizer (VCM) | Concern on children and pregnant women wellbeing i.e., create awareness on exclusive breastfeeding and other child health related matters |
| 35. | Ward development committee** | Work with health facility on health matters (link between health facility and hospital) |
| 36. | Volunteer village health workers (VVHW) | Volunteer community mobilisers are on paid role and their role is to pass information such immunization, exclusive breastfeeding to members of the community  Or to organize mothers to bring their children out for immunization. These are mostly women because women can enter into people’s houses unlike men |
| 37. | Health Ambassador | Paid role, similar to VVHW (TBC by Funmilayo) |
| 38. | SHUDA – Shuwarin development group | Helps to maintain good sanitation within the community. It is a type of village development committee (VDC) |
| 39. | President | Grant permission to any international body coming into the community |
| 40. | Buhari (President) support group | Render financial help to parents of which they use to cater for their children wellbeing |
| 41. | Women group | Groups of women within the community that give both financial and emotional support to parents of sick children. NOTE: not all wards mentioned this group |
| 42. | Motorcycle (Bike) support group | They transport pregnant women to hospitals on roads cars cannot access |
| 43. | Safe delivery committee (Haihuwa lafiya) | They transport pregnant women in emergency cases to the hospital |
| 44. | Village association | Identified on map, but no clear definition given. |
| 45. | School base management committee | Identified on map, but no clear definition given. |
| 46. | National union of road transportation (NURTW) | Identified on map, but no clear definition given. |
| 47. | Polio team | Give vaccines to children |
| 48. | Malaria team | Distribute LLINs and malaria drugs |
| 49. | Health educators | Similar to VVHW in terms of role – TBC if paid/unpaid role. |
| 50. | Water resource group | Help to bring water into the community |
| 51. | Rich people | Identified on map, but no clear definition given. |
| 52. | Solar power | Identified on map, but no clear definition given. |
| 53. | Native doctors/Traditional healers | Help to cure some child illnesses e.g., palli-palli |
| 54. | Traditional birth attendance | Take delivery, work hand in hand with doctors to administer basic treatment |
| 55. | Committee of hospital | They serve as link between the hospital and the community |
| 56. | MNCH2 | Give free drugs to under five children and pregnant women |
| 57. | Town crier | They help to pass crucial information within community |
| 58. | Youth association | Donates in cash and kind to patients i.e., donates blood and help financially |
